# Comparative effectiveness of hypoxia-inducible factor prolyl hydroxylase inhibitors versus erythropoiesis-stimulating agents on prognosis in non-dialysis chronic kidney disease: a propensity-matched cohort study

**DOI:** 10.1080/0886022X.2025.2592442

**Published:** 2025-12-04

**Authors:** Yi-Chou Hou, Chia-Lin Wu, Wei-Cheng Tseng, Joshua Wang, Kuo-Cheng Lu, Cai-Mei Zheng, Jia-Sin Liu, Ko-Lin Kuo, Der-Cherng Tarng

**Affiliations:** ^a^ Department of Internal Medicine, Division of Nephrology, Cardinal Tien Hospital, New Taipei City, Taiwan; ^b^ School of Medicine, Fu Jen Catholic University, New Taipei City, Taiwan; ^c^ Department of Post-Baccalaureate Medicine, College of Medicine, National Chung Hsing University, Taichung, Taiwan; ^d^ School of Medicine, Chung Shan Medical University, Taichung, Taiwan; ^e^ Division of Nephrology, Department of Internal Medicine, Changhua Christian Hospital, Changhua, Taiwan; ^f^ Department of Nephrology, Changhua Christian Hospital, Changhua City, Taiwan; ^g^ Division of Nephrology, Department of Medicine, Taipei Veterans General Hospital, Taipei, Taiwan; ^h^ School of Medicine, College of Medicine, National Yang Ming Chiao Tung University, Taipei, Taiwan; ^i^ Department of Research, Taipei Tzu Chi Hospital, Buddhist Tzu Chi Medical Foundation, New Taipei City, Taiwan; ^j^ School of Biomedical Sciences, Queensland University of Technology, Brisbane, Queensland, Australia; ^k^ Division of Nephrology, Department of Medicine, Taipei Tzu Chi Hospital, Buddhist Tzu Chi Medical Foundation, New Taipei City, Taiwan; ^l^ School of Medicine, Tzu Chi University, Hualien, Taiwan; ^m^ Division of Nephrology, Department of Internal Medicine, Shuang Ho Hospital, Taipei Medical University, New Taipei City, Taiwan; ^n^ Department of Internal Medicine, School of Medicine, College of Medicine, Taipei Medical University, Taipei, Taiwan; ^o^ Graduate Institute of Clinical Medicine, College of Medicine, Taipei Medical University, Taipei, Taiwan; ^p^ School of Post-Baccalaureate Chinese Medicine, Tzu Chi University, Hualien, Taiwan; ^q^ Institute of Clinical Medicine, School of Medicine, National Yang Ming Chiao Tung University, Taipei, Taiwan

**Keywords:** Chronic kidney disease, renal anemia, hypoxia-inducible factor prolyl hydroxylase inhibitors, erythropoiesis-stimulating agents, sepsis

## Abstract

Renal anemia in patients with stage-IV chronic kidney disease (CKD) is commonly treated with erythropoiesis-stimulating agents (ESAs), which are effective but associated with cardiovascular risks, variable responsiveness, and inflammatory complications. Hypoxia-inducible factor prolyl hydroxylase inhibitors (HIF-PHIs) have recently emerged as an alternative therapy that enhances endogenous erythropoietin production and improves iron metabolism. This retrospective cohort study utilized the TriNetX Global Collaborative Network to compare clinical outcomes between HIF-PHI and ESA users in non-dialysis stage IV CKD patients (estimated glomerular filtration rate 15–30 mL/min/1.73 m^2^). After 1:1 propensity score matching, 493 patients were included in each group and followed for up to three years. HIF-PHI therapy was associated with significantly lower all-cause mortality (hazard ratio [HR] 0.39; 95% confidence interval [CI] 0.26–0.58; *p* < 0.0001) and reduced sepsis risk (HR 0.32; 95% CI 0.14–0.74; *p* = 0.01) compared with ESA therapy. Subgroup analyses demonstrated consistent mortality benefit across major comorbidities, with the greatest advantage observed in patients with ferritin 100–299 ng/mL (HR 0.41; 95% CI 0.23–0.74; *p* = 0.002) and in comparisons against short-acting ESAs (HR 0.55; *p* = 0.0304). No survival difference was observed between HIF-PHI and long-acting ESA users, indicating that the observed benefit of HIF-PHIs was driven exclusively by differences versus short-acting ESAs. These findings suggest that HIF-PHIs may offer a safer and more physiologically adaptive approach to correcting anemia in advanced CKD, particularly when compared with short-acting ESA therapy. In contrast, long-acting ESAs achieve outcomes comparable to those of HIF-PHIs.

## Introduction to chronic kidney disease and its complications

Chronic kidney disease (CKD) is a progressive condition characterized by a persistent decline in glomerular filtration rate and structural kidney damage [1]. CKD patients are at risk of developing multiple comorbidities, including heart failure, coronary artery disease (CAD), and renal osteodystrophy, which result from fluid overload, hypertension, and dysregulated mineral metabolism [2–4]. Among these complications, renal anemia is a major concern, significantly impacting patients’ quality of life and increasing cardiovascular morbidity and mortality [5,6]. Effective management of renal anemia is essential in CKD care to reduce the burden of these complications.

Renal anemia is primarily caused by insufficient erythropoietin (EPO) production due to kidney dysfunction, along with iron dysregulation and chronic inflammation. The standard treatment involves erythropoiesis-stimulating agents (ESAs), which stimulate red blood cell production and improve hemoglobin levels [1,7]. ESAs have been widely used in CKD patients, particularly those on dialysis, as they are effective in correcting anemia and reducing the need for blood transfusions. However, their use is associated with several limitations, including increased risks of cardiovascular events, hypertension, and thromboembolism with hemoglobin levels higher than 12 g/dL [8,9]. Additionally, ESA responsiveness varies among patients, and maintaining optimal hemoglobin levels remains challenging [10]. High doses of erythropoietin observed in ESA-associated clinical trials have been indicative of ESA hypo-responsiveness, which is associated with poorer outcomes, including increased mortality [11,12]. Recent clinical studies have raised concerns about the potential overuse of ESAs and the need for alternative therapies that can address these limitations.

Hypoxia-inducible factor proxyl hydroxylase inhibitor (HIF-PHI) represents a novel therapeutic approach for renal anemia. HIF-PHI, such as roxadustat, vadadustat, and daprodustat, work by activating endogenous EPO production in response to hypoxia, promoting iron kinetics, and maintaining erythropoiesis under inflammatory status [13]. In contrast to ESA, HIF-PHI provided a sustained physiologic concentration of serum erythropoietin [14]. These drugs have gained significant attention, which has recognized the discovery of HIF’s role in oxygen sensing [15]. Clinical trials have demonstrated the efficacy of HIF-PHI in treating anemia among end-stage renal disease (ESRD) patients undergoing dialysis [16]. However, their clinical outcomes differ among studies focusing on nondialysis CKD patients, and further research is needed to determine their long-term safety and effectiveness in this population [17].

The draft of Kidney Disease: Improving Global Outcomes (KDIGO) listed HIF-PHI as the second-line therapy for renal anemia due to safety issues [18], but the real-world experience for comparing the HIF-PHI and ESAs is lacking. This study aims to utilize the TrinetX database to compare the efficacy and safety of HIF-PHI versus ESAs in real-world settings. By analyzing large-scale clinical data, we seek to determine whether HIF-PHI offers superior benefits over ESAs, particularly in nondialysis CKD patients where treatment options remain uncertain.

## Materials and methods

### Study design and ethics

This retrospective cohort study was conducted using data from TriNetX, a global federated health research network comprising electronic medical records (EMRs) from 141 healthcare organizations. The TriNetX Global Collaborative Network offers access to anonymized data on diagnoses, laboratory results, medications, and procedures [19]. The analysis was executed on February 3, 2025, covering data collected from January 1, 2020, through December 31, 2024. The study was approved by the Institute of Research Board of Taipei Tzu-Chi Hospital, New Taipei City, Taiwan (14-IRB039). The TriNetX database contains only de-identified patient information; the requirement for informed consent was waived. All research procedures were conducted in accordance with the principles stated in the *Declaration of Helsinki*.

### Cohort definition and study population

**Figure 1.**
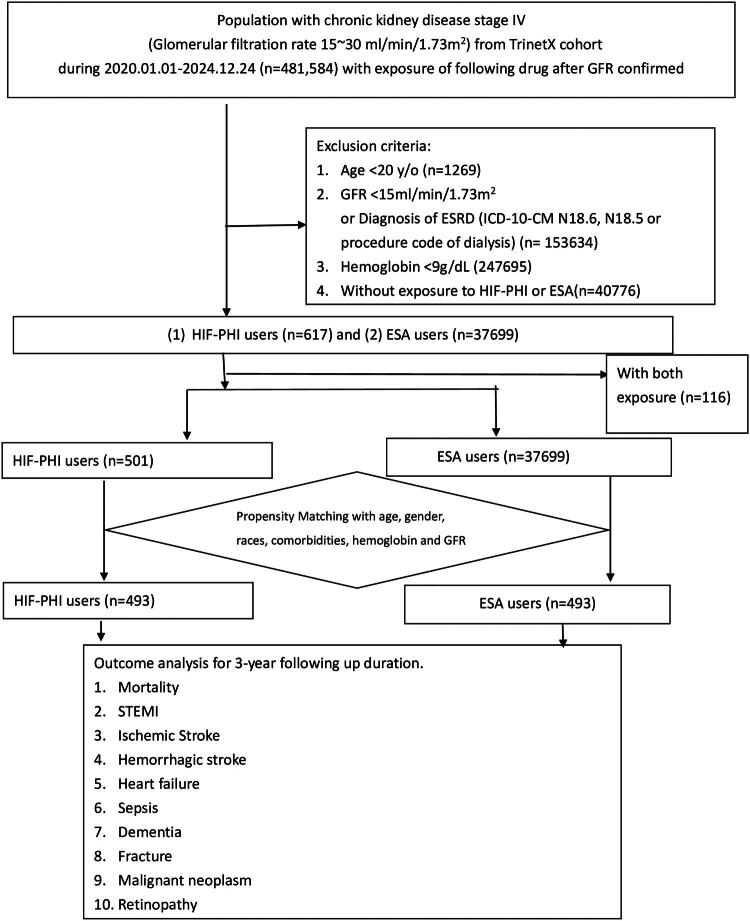
Flow chart of study cohort selection and matching process. Patients with stage IV chronic kidney disease (CKD, eGFR 15–30 mL/min/1.73m^2^) were identified from the TriNetX platform between Jan 1, 2020, and Dec 24, 2024. After applying exclusion criteria, 501 HIF-PH inhibitor users and 37,699 ESA users were identified. Following 1:1 propensity score matching, 493 pairs were included for outcome analysis.

To discrete the patients with dialysis, the subjects with GFR 15–30 mL/min were enrolled. The index date was defined as the first prescription of either treatment after confirmation of stage IV. Outcomes were tracked for 3 years beginning 1 day after the index event. The flowchart of enrollment is listed in Figure 1. Patients with stage-IV chronic kidney disease (CKD), defined by an estimated glomerular filtration rate (eGFR) of 15–30 mL/min/1.73m^2^ recorded on at least two occasions, were included. Eligible patients were required to have a hemoglobin level ≥9 g/dL and be at least 20 years of age. The subjects with a history of dialysis or ESRD (ICD-10-CM N18.5, N18.6, or relevant CPT codes for dialysis, listed in Supplement 1) were excluded. The study compared two treatment cohorts:

**Figure 2.**
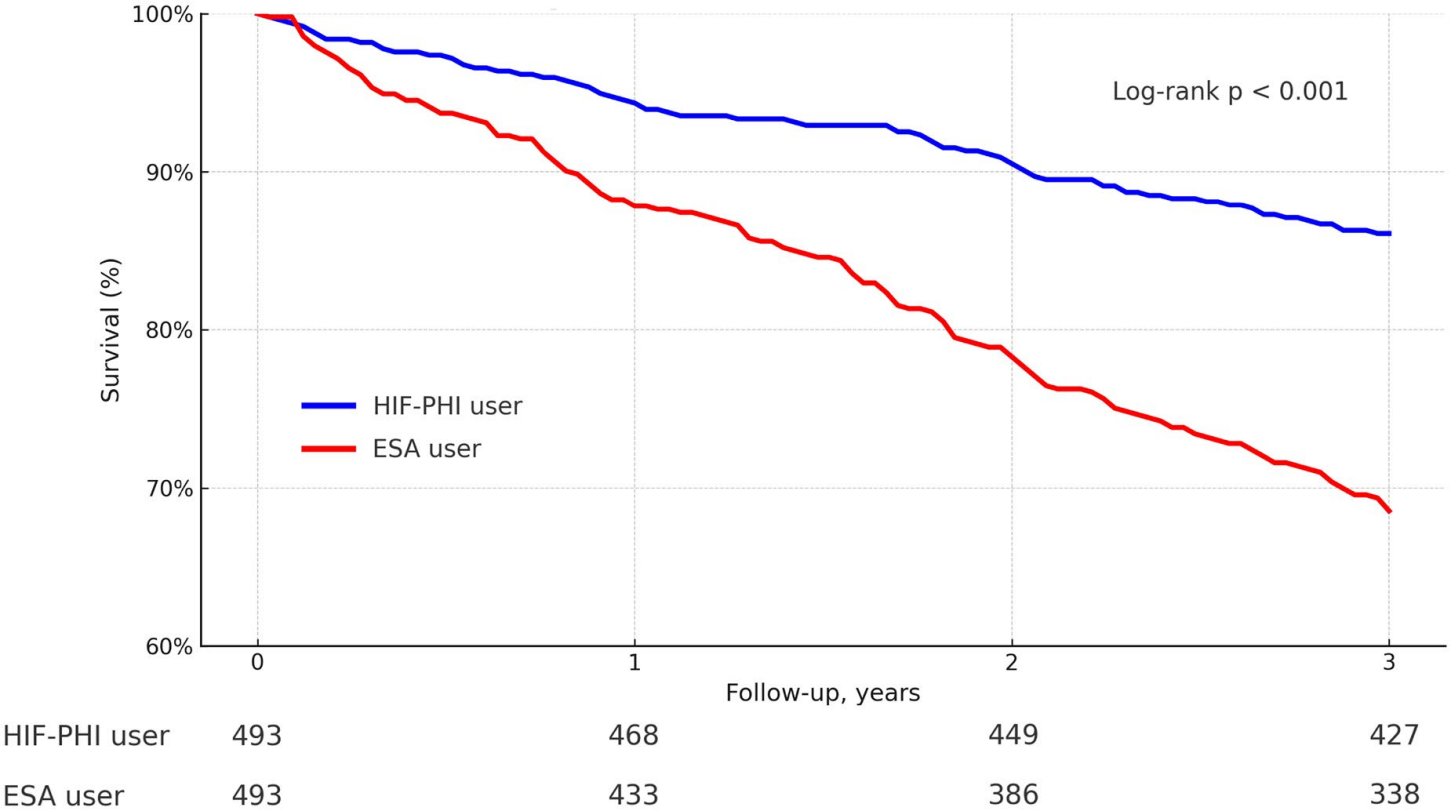
Kaplan–Meier curve comparing all-cause mortality between HIF-PHI and ESA users. Propensity score–matched patients with stage IV CKD were followed for up to 3 years. HIF-PHI users (blue line) showed significantly lower mortality compared to ESA users (red line) (log-rank *p* < 0.0001).

**Figure 3.**
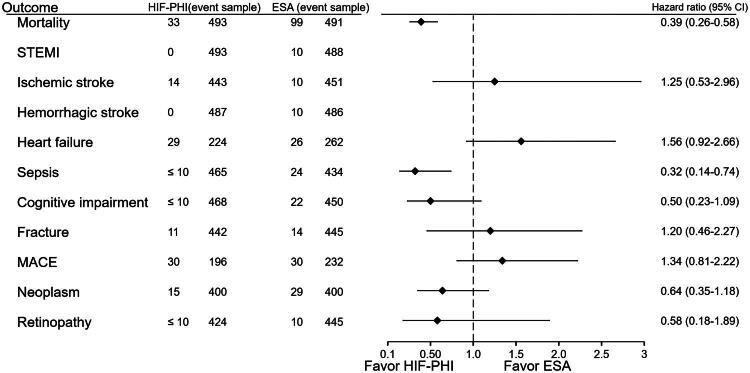
Hazard ratios for major clinical outcomes: HIF-PHI vs ESA. Adjusted hazard ratios for all-cause mortality, sepsis, cardiovascular events, malignancy, and cognitive impairment were calculated after propensity score matching. HIF-PHI use was associated with significantly reduced risks of death (HR 0.39) and sepsis (HR 0.32).

- HIF-PHI Group: Patients treated with hypoxia-inducible factor prolyl hydroxylase inhibitors (daprodustat, vadadustat, roxadustat), without prior use of ESAs as the supplement 1).
- ESA Group: Patients treated with ESAs (epoetin alfa, darbepoetin alfa, methoxy polyethylene glycol-epoetin beta) without previous exposure to HIF-PHI (as supplement 1).

Patients receiving both treatments or undergoing dialysis to treatment initiation were excluded. Patients without exposure to HIF-PHI or ESA were excluded as well.

### Propensity score matching

To reduce confounding, 1:1 propensity score matching was conducted using 16 covariates, including demographics (age, sex, ethnicity), comorbidities (diabetes mellitus, heart failure, ischemic heart disease, cerebrovascular disease, chronic lower respiratory disease, glomerular disease), and laboratory values (hemoglobin and eGFR levels). Matching yielded 496 patients in each group (HIF-PHI vs ESA), ensuring balanced baseline characteristics for downstream analysis.

### Covariates and baseline characteristics

Baseline characteristics included demographics, comorbidities, medication use (e.g. ACE inhibitors, beta-blockers, calcium channel blockers), and laboratory values (e.g., hemoglobin, sodium, creatinine, ferritin, GFR, BMI).

### Outcomes and definitions

The primary outcome was all-cause mortality. Secondary outcomes included:

- Cardiovascular events: Heart failure, ST-segment elevation myocardial infarction, major adverse cardiovascular events (MACE), stroke (ischemic and hemorrhagic)
- Infectious complications: Sepsis
- Other events: Fracture, malignancy, retinopathy, dementia, and hospitalization

Outcome definitions followed ICD-10-CM codes (detailed in Supplementary Table 1). Patients with a documented outcome before the index date were excluded from that outcome analysis. All analyses were performed on the TriNetX analytics platform, with a time window spanning 1,095 days post-index event.

Subgroup analysis: Comparison of HIF-PHI users with long- and short-acting ESA on mortality

To explore potential differences in survival outcomes according to erythropoiesis-stimulating agent (ESA) pharmacokinetics, we established three treatment cohorts based on drug exposure among patients with stage IV chronic kidney disease (CKD), defined by an estimated glomerular filtration rate (eGFR) of 15–30 mL/min/1.73 m^2^. The short-acting ESA group comprised patients prescribed epoetin alfa or epoetin beta (RxNorm 1816044, 1816005) with eGFR 15–30 mL/min/1.73 m^2^ who were not receiving dialysis [20]. These agents represent the conventional, short half-life erythropoietin analogs requiring two- to three-times-weekly dosing. The long-acting ESA group included patients prescribed darbepoetin alfa or methoxy polyethylene glycol-epoetin beta (RxNorm 2583538, 729986) with eGFR 15–30 mL/min/1.73 m^2^ and no record of dialysis. Each cohort required two or more prescription encounters for the qualifying agent and a laboratory-confirmed eGFR value within the defined range to treatment initiation. Patients with any dialysis-related procedure codes (CPT 90935–90999) or an end-stage kidney disease diagnosis before the index date were excluded to ensure inclusion of non-dialysis CKD subjects. 1:1 propensity-score matching (PSM) was performed with matching variables as previous section, along with concurrent medication use, including angiotensin-converting enzyme inhibitors (ACE inhibitors) and angiotensin II receptor blockers (ARBs), identified through RxNorm or ATC coding systems.

### Statistical analysis

Values are reported as means ± SD or percentages. Statistical comparisons were performed using t-tests for categorical variables and chi-square tests. Survival analysis was performed using the Kaplan–Meier method, and survival differences were assessed using the log-rank test. Hazard ratios (HRs) and 95% confidence intervals (CIs) were estimated using Cox proportional hazards models. Hazard ratios were calculated in TriNetX for binary outcomes. A two-tailed test with *p*-value <0.05 was considered statistically significant.

## Results

### Patients characteristics

A total of 38,200 patients with advanced CKD were analyzed. We identified 38,200 individuals using HIF-PHI and ESA. 501 subjects were HIF-PHI users, and 37,699 subjects were ESA users. The prevalence of HIF-PHI users was 1.3% (501 of 38,200). The median follow-up period for the entire cohort was 1.5 years. The 25th percentile indicates a duration of 0.75 years, while the 75th percentile extends to 2.25 years. Table 1 illustrates the demographic results. After matching, the mean age was 76.8 ± 10.3 years in the HIF-PHI group and 76.7 ± 8.7 years in the ESA group (*p* = 0.797). The percentage of sex and comorbidities, such as hypertension or diabetes mellitus, was similar between groups. In the HIF-PHI group, the percentage of White individuals was 0% both before and after propensity score matching, whereas individuals with unknown ethnicity accounted for 100%. We searched for the origin of the database. All subjects originated from the Asia-Pacific (APAC) collaborative network. None of the subjects originated from the United States or the European Collaborative Network. Table 1 also demonstrated the biochemical and hematologic Comparisons. In terms of medication use, diuretics were prescribed to 46.7% of HIF-PHI users and 58.2% of ESA users (*p* < 0.001), beta-blockers to 40.6% and 54.2%, respectively (*p* < 0.001), and lipid-lowering agents to 42.3% and 51.4% (*p* = 0.002). Conversely, SGLT2 inhibitors were more frequently used in the HIF-PHI group (24.7%) compared to the ESA group (26.3%), though the difference was not statistically significant (*p* = 0.533). Regarding laboratory parameters, the HIF-PHI group had significantly higher serum sodium levels (139.0 ± 3.8 vs 138.3 ± 3.9 mmol/L, *p* = 0.003), hemoglobin (9.9 ± 1.7 vs 9.6 ± 1.5 g/dL, *p* = 0.002), and total cholesterol (162.5 ± 50.0 vs 140.2 ± 49.6 mg/dL, *p* < 0.001). The ESA group exhibited higher HbA1c (6.5 ± 1.3 vs 6.1 ± 1.0%, *p* = 0.001), BMI (26.6 ± 6.4 vs 22.1 ± 4.1 kg/m^2^, *p* < 0.001), and ferritin (348.1 ± 504.2 vs 246.5 ± 314.6 ng/mL, *p* = 0.003). LDL cholesterol, glucose, and GFR values also favored the ESA group, though some differences did not reach statistical significance.

**Table 1.** The demographics, comorbidities, medications and laboratory data in hypoxia-inducible factor prolyl hydroxylase inhibitor(HIF-PHI) and erythropoiesis-stimulating agents(ESA) users before and after propensity-score matching.

	Before propensity-score matching				After propensity-score matching			
	HIF-PHI users	ESA users	p-value	SMD	HIF-PHI users	ESA users	p-value	SMD
n	501	37,699			493	493		
Age at Index, years old	77.8 ± 9.0	68.5 ± 12.5	<0.001	0.855	76.8 ± 10.3	76.7 ± 8.7	0.797	0.016
Sex								
Male, n (%)	333 (54.0)	34,995 (51.0)	0.212	0.051	251 (54.3)	333 (54.3)	>0.99	<0.001
Female, n (%)	284 (46.0)	31,434 (47.0)	0.928	0.004	242 (49.1)	244 (49.5)	0.899	0.008
Ethnicity								
Unknown, n (%)	501 (>99.9)	27,297 (40.0)	<0.001	1.732	493 (>99.9)	493 (>99.9)	>0.99	<0.001
White, n (%)	0 (<0.1)	31,750 (46.4)	<0.001	1.323	0 (<0.1)	0 (<0.1)	>0.99	<0.001
Comorbidities								
Glomerular diseases, n (%)	45 (9.0)	2,936 (7.9)	0.391	0.037	45 (9.1)	43 (8.7)	0.823	0.014
Other forms of heart disease, n (%)	237 (47.3)	18,969 (51.3)	0.076	0.080	230 (46.7)	230 (46.7)	>0.99	<0.001
Hypertensive diseases, n (%)	181 (36.1)	23,463 (63.4)	<0.001	0.568	179 (36.3)	188 (38.1)	0.553	0.038
Ischemic heart diseases, n (%)	102 (16.5)	23,130 (34.0)	<0.001	0.410	88 (17.8)	83 (16.8)	>0.99	<0.001
Cerebrovascular diseases, n (%)	58 (11.6)	5,803 (15.7)	0.012	0.120	58 (11.8)	54 (11.0)	0.674	0.027
Heart failure, n (%)	203 (40.5)	12,337 (33.4)	0.001	0.149	196 (39.8)	202 (40.1)	0.697	0.025
Diabetes mellitus, n (%)	151 (30.1)	15,605 (42.2)	<0.001	0.253	150 (30.4)	154 (31.2)	0.783	0.018
Medications								
Calcium Channel Blockers, n (%)	270 (53.9)	19,359 (52.3)	0.487	0.031	266 (54.0)	240 (48.7)	0.098	0.106
Angiotensin II Inhibitor/ACE Inhibitors, n (%)	289 (57.7)	20,411 (55.1)	0.415	0.003	284 (57.6)	290 (58.8)	0.474	0.010
NSAID, n (%)	63 (12.0)	6,524 (17.0)						
Diuretics, n (%)	237 (47.3)	25,427 (68.8)	<0.001	0.445	230 (46.7)	287 (58.2)	<0.001	0.233
Antilipemic agents, n (%)	221 (44.1)	20,783 (56.2)	<0.001	0.244	261 (42.3)	315 (51.4)	0.002	0.177
Beta Blockers, n (%)	206 (41.1)	23,713 (64.1)	<0.001	0.473	200 (40.6)	267 (54.2)	<0.001	0.275
Iron, n (%)	164 (32.7)	12,487 (33.8)	0.628	0.022	161 (32.7)	128 (26.0)	0.021	0.147
SGLT2i, n (%)	133 (26.5)	2,036 (5.4)	<0.001	0.103	122 (24.7)	130 (26.3)	0.533	0.028
Short-acting ESA		2,9208						
Long-acting ESA (*n* = 14204)								
Darbepoetin alfa, n(% of long-acting ESA)		1,252 6(88)						
Methoxy polyethylene glycol-epoetin beta n(% of long-acting ESA)		2,575 (18)						
Laboratory Data								
Sodium (mmol/L), mean (SD)	139.0 ± 4.1	137.7 ± 4.8	<0.001	0.281	139.0 ± 3.8	138.3 ± 3.9	0.003	0.196
Potassium (mmol/L), mean (SD)	4.4 ± 0.7	4.3 ± 0.7	0.025	0.125	4.4 ± 0.7	4.4 ± 0.7	0.290	0.069
Chloride (mmol/L), mean (SD)	105.0 ± 5.7	102.8 ± 9.6	<0.001	0.284	104.9 ± 5.3	104.1 ± 5.9	0.053	0.133
Bicarbonate (mmol/L), mean (SD)	25.2 ± 4.2	23.1 ± 4.8	0.364	0.135	22.6 ± 3.9	23.0 ± 4.8	0.634	0.076
Urea nitrogen (mg/dL), mean (SD)	43.3 ± 21.0	48.6 ± 26.0	<0.001	0.224	41.4 ± 19.9	42.4 ± 23.4	0.604	0.038
Creatinine (mg/dL), mean (SD)	2.7 ± 1.5	3.4 ± 8.0	0.107	0.121	2.8 ± 1.7	3.0 ± 1.7	0.051	0.131
Glomerular filtration rate/1.73 m^2^ [creatinine-based formula; MDRD)	20.7 ± 10.9				20.6 ± 10.9	21.6 ± 15.8	0.252	0.075
Glucose (mg/dL), mean (SD)	125.9 ± 46.8	124.3 ± 56.0	0.615	0.030	131.1 ± 52.7	125.0 ± 51.9	0.090	0.117
Calcium (mg/dL), mean (SD)	8.7 ± 0.7	8.6 ± 0.9	0.386	0.056	8.7 ± 0.7	8.6 ± 1.0	0.082	0.125
Phosphate (mg/dL), mean (SD)	3.8 ± 1.0	4.1 ± 1.4	0.003	0.257	3.9 ± 1.0	3.9 ± 1.3	0.342	0.081
Hemoglobin (g/dL), mean (SD)	9.8 ± 1.6	8.8 ± 1.6	<0.001	0.593	9.9 ± 1.7	9.6 ± 1.5	0.002	0.205
<9 g/dL	258 (51.5%)	25,362 (68.6%)	<0.001	0.354	258 (52.0%)	247 (49.8%)	0.485	0.044
9-11 g/dL	410 (81.8%)	30,649 (82.9%)	0.540	0.027	407 (82.1%)	404 (81.5)	0.805	0.016
>11 g/dL	356 (71.1%)	24,144 (65.3%)	0.007	0.124	353 (71.2%)	362 (73.0%)	0.524	0.040
Microalbumin/creatinine ration (mg/g)	369 ± 380	809 ± 1612	0.388	0.376	369 ± 380	852 ± 1293	0.271	0.507
Ferritin (ng/mL)	208.5 ± 314.0	586.8 ± 1799.4	0.003	0.293	210.4 ± 317.6	343.8 ± 517.3	0.002	0.311
Albumin (g/dL)	3.3 ± 0.7	3.2 ± 0.7	0.037	0.015	3.3 ± 0.7	3.3 ± 0.7	0.521	0.053
Total protein (g/dL)	6.46 ± 0.79	6.62 ± 1.63	0.205	0.121	5.96 ± 0.56	6.20 ± 0.65	0.407	0.379
C-reactive protein (mg/dL)	21.6 ± 48.1	46.2 ± 68.5	<0.001	0.505	23.0 ± 28.5	42.5 ± 42.5	0.044	0.964

Abbreviations: SD: standard deviation. SMD: Standardized mean difference. NSAID: Non-steroidal anti-inflammatory drugs, SGLT2 inhibitor: Sodium-glucose Cotransporter 2 Inhibitor.

### Mortality analysis (Figure 2)

Kaplan-Meier analysis showed a significantly lower all-cause mortality in the HIF group compared to the ESA group, with a highly significant log-rank test (*p* < 0.0001), indicating improved survival among HIF users.

### Hazard ratios for major outcomes (Figure 3)

The comparative analysis of major clinical outcomes showed that HIF-PHI inhibitors were associated with a substantially lower risk of all-cause mortality, with a HR of 0.39 (95% CIs: 0.26–0.58). Similarly, the risk of sepsis was significantly lower in the HIF group (HR: 0.32; 95% CIs: 0.14–0.74). While other outcomes, such as heart failure, major adverse cardiovascular events (MACE), malignancies, and cognitive decline, also trended toward benefit in the HIF group, these did not uniformly reach statistical significance. For the outcome of renal replacement therapy, no HIF-PHI users (0/344) experienced the event of interest, whereas 10 events occurred among ESA users (10/330). This difference reached statistical significance (*p* = 0.022). HR was not performed by the platform due to a low case number.

### Subgroup analysis by comorbidities (Figures 4 and 5)

**Figure 4.**
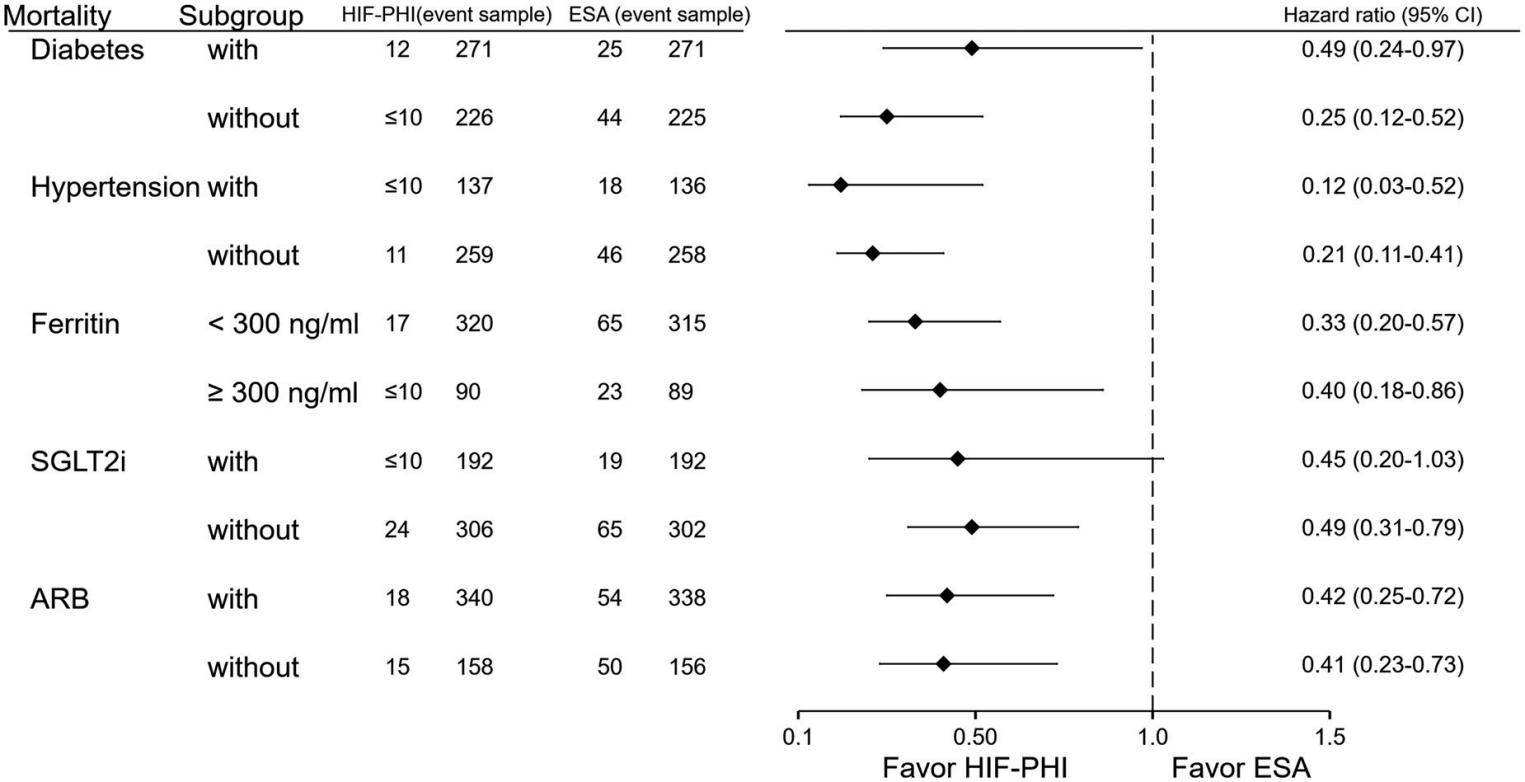
Subgroup analysis of all-cause mortality comparing HIF-PHI and ESA users. Mortality hazard ratios were stratified by comorbidities (diabetes, hypertension) and showed consistent benefit for HIF-PHI in all subgroups. Notably, the HR was 0.25 in patients without diabetes (*p* < 0.0001).

**Figure 5.**
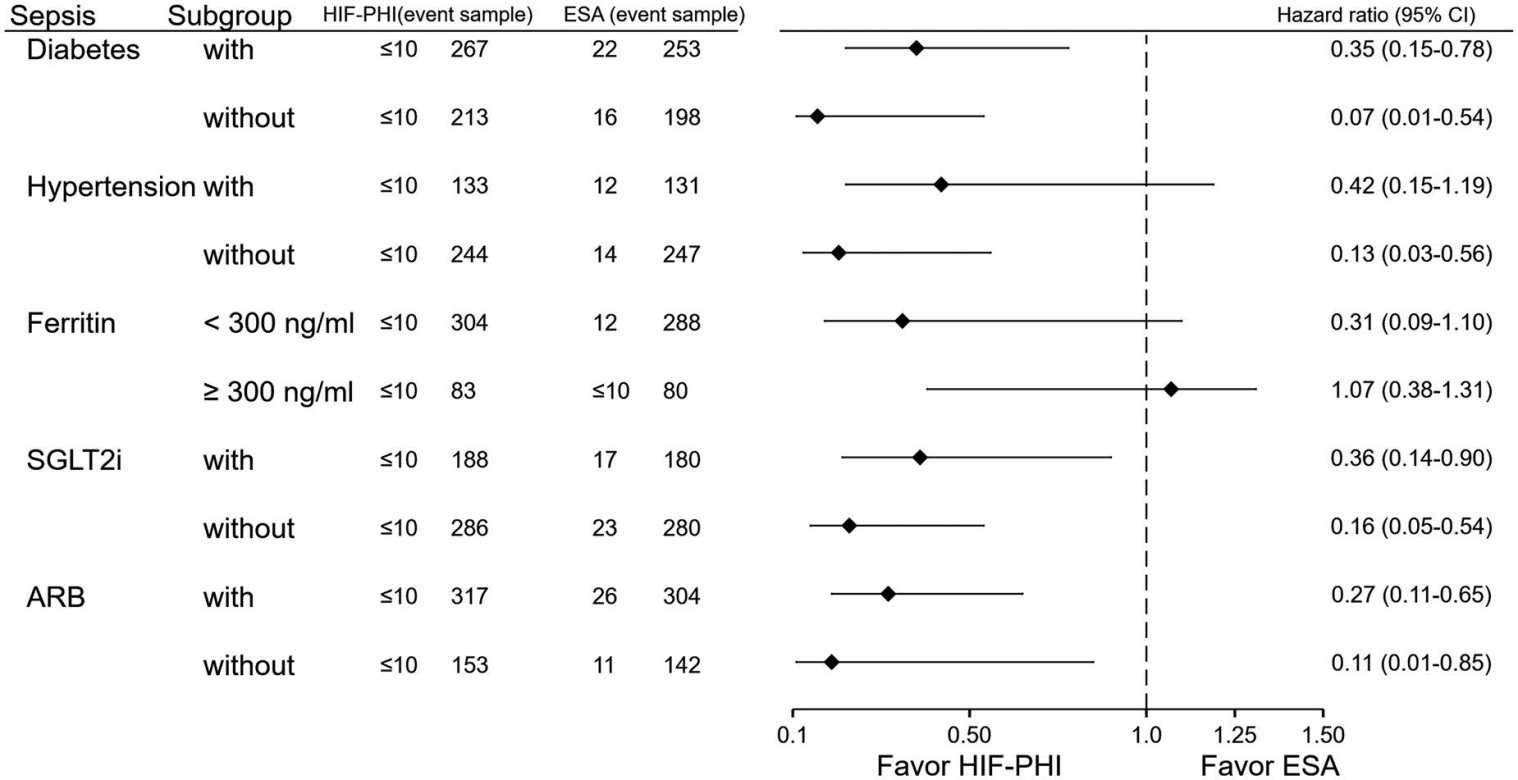
Subgroup analysis of sepsis risk in HIF-PHI vs ESA users. Most comorbidity subgroups showed lower sepsis risk with HIF-PHI use, although the difference in hypertensive patients did not reach statistical significance (HR: 0.42, *p* = 0.09).

Subgroup analyses stratified by comorbid conditions consistently showed reduced mortality (Figure 4) and sepsis (Figure 5) risks among HIF users. In patients with diabetes mellitus (DM), mortality risk was nearly halved (HR: 0.49; 95% CI: 0.24–0.97; *p* = 0.04). In those without diabetes, the protective effect was more pronounced (HR: 0.25; 95% CI: 0.12–0.52; *p* < 0.0001). HIF-PHI users also had significantly lower mortality in both hypertensive (HR: 0.12; 95% CI: 0.03–0.52; *p* = 0.0006) and non-hypertensive (HR: 0.21; 95% CI: 0.11–0.41; *p* < 0.0001) patients. Stratification by ferritin showed consistent benefit: patients with ferritin <300 ng/mL had a HR of 0.33 (95% CI: 0.20–0.57; *p* < 0.0001), and those with ferritin ≥ 300 ng/mL had a HR of 0.40 (95% CI: 0.18–0.86; *p* = 0.02).

For sepsis, only patients with hypertension showed no significant benefit from HIF-PHI (HR: 0.42; 95% CI: 0.15–1.19; *p* = 0.09). Ferritin level was not associated with sepsis risk. HIF-PHI use was linked to lower sepsis risk in other subgroups.

### Ferritin-stratified analysis (Figure 6)

Further analysis based on serum ferritin levels revealed that patients with intermediate ferritin levels (100–299 ng/mL), the mortality in this group was reduced (HR: 0.41, 95%CI 0.23–0.74; *p* = 0.002), while the risks of sepsis (HRs: 0.09, 95%CI 0.012–0.69, *p* = 0.004) and dementia (HR = 0.18, 95%CI 0.04–0.82; *p* = 0.01)were also markedly lower.

In patients with ferritin <100 ng/mL, the benefit on mortality did not differ between HIF-PH inhibitor or ESA users. Ischemic stroke incidence was significantly reduced (HR: 0.19, 95%CI 0.04–0.81; *p* = 0.012), along with lower risks of sepsis (HR: 0.20, 95%CI 0.05–0.91; *p* = 0.021) and fracture (HR: 0.35, 95%CI 0.12–1.03; *p* = 0.047). Mortality also trended lower, though the result did not reach statistical significance (HR: 0.72, 95%CI 0.39–1.35; *p* = 0.278). Post-treatment variation of hemoglobin, ferritin, and potassium levels was compared between the 2 groups before matching. Before matching, the post-treatment ferritin level was lower in the HIF group (264.7 ± 657.1 ng/mL) compared with the ESA group (587.0 ± 2,613.6 ng/mL; *p* = 0.0096). Post-treatment hemoglobin levels were comparable between the HIF group (11.04 ± 1.44 g/dL) and the ESA group (11.06 ± 1.86 g/dL; *p* = 0.7724). Post-treatment potassium was slightly higher in the HIF group (4.44 ± 0.75 mmol/L) than in the ESA group (4.33 ± 0.64 mmol/L; *p* < 0.0001). Regarding adverse events, the incidence of hyperkalemia was lower in the HIF group (9.70%) than in the ESA group (13.79%), with a risk ratio of 0.703 (95% CI 0.566–0.874) and an odds ratio of 0.671 (95% CI 0.527–0.854). Post-matching analysis was not feasible because the patient count was too small to generate detailed results.

**Figure 6.**
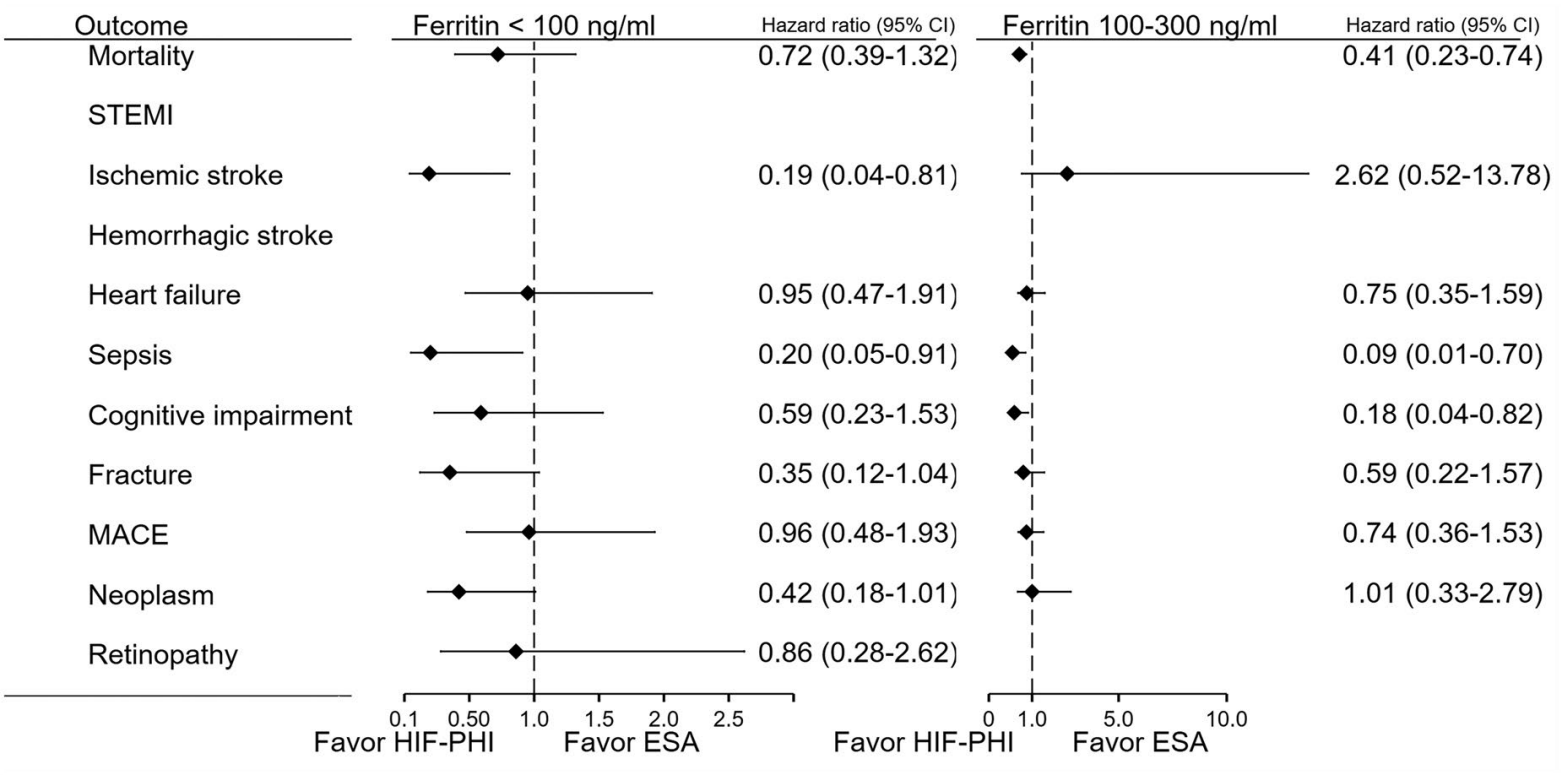
Outcomes stratified by ferritin level: comparison of HIF-PHI and ESA. Outcomes were analyzed by ferritin level: (A) <100 ng/mL, (B) 100–299 ng/mL. HIF-PHI users in group B had significantly lower mortality (HR 0.41), sepsis (HR 0.09), and dementia (HR 0.18).

#### The comparison of HIF-PHI with long- and short-acting ESA on mortality

The Supplement Table 2 summarizes the comparative survival analyses between patients receiving hypoxia-inducible factor prolyl hydroxylase inhibitors (HIF-PHIs) and those treated with ESAs. After 1:1 propensity-score matching by the previous factors along with ACEi/ARB use, three separate analyses were performed comparing HIF-PHI with [1] all ESA users [2], short-acting ESA users, and [3] long-acting ESA users. The table displays patient counts before and after matching, event numbers, survival probabilities at the end of follow-up, log-rank statistics, and corresponding HRs with 95% CIs.HIF-PHI therapy was associated with a significantly lower mortality risk compared with overall ESA use (HR = 0.533, *p* = 0.0063). The advantage persisted when compared specifically with short-acting ESAs (HR = 0.554, *p* = 0.0304). Outcomes were similar between HIF-PHI and long-acting ESA groups (HR = 1.172, *p* = 0.9585), indicating comparable long-term survival. To compare survival among long-acting ESAs, Kaplan–Meier and Cox analyses were conducted for darbepoetin alfa and methoxy polyethylene glycol-epoetin beta. Compared with darbepoetin, HIF-PHI therapy showed higher survival at follow-up (74.3% vs 56.0%; log-rank χ^2^ = 8.812, *p* = 0.0030) with a HR of 0.54 (95% CI 0.36–0.82). The proportional-hazards assumption was met (*p* = 0.57). In contrast, survival did not differ between HIF-PHI and methoxy polyethylene glycol-epoetin beta users (76.9% vs 75.2%; χ^2^ = 0.095, *p* = 0.76; HR 1.09, 95% CI 0.64–1.84, *p* = 0.14). Overall, these findings suggest that the survival benefit of HIF-PHIs is primarily driven by differences versus short-acting ESAs, whereas long-acting ESAs achieve outcomes comparable to HIF-PHI therapy.

## Discussion

From the retrospective global cohort, the HIF-PHI was associated with a benefit in overall survival in the subjects with GFR 15–30 mL/min. The hazard ratio for sepsis was lower in the HIF-PHI users than in the ESA users. The protective effect was significant during the subgroup analysis, and the ferritin level was associated with the protective efficacy of HIF-PHI. The development of malignancy or retinopathy was similar between HIF-PHI users and ESA. ESA has been regarded as the cornerstone treatment for renal anemia. For dialysis-dependent CKD, the ESA replenishes the production of red blood cells in the bone marrow and replaces the necessity of repeated blood transfusions [21]. The clinical trials of ESA focused on the targeted level of the hemoglobin, and the target Hb level was associated with the cardiovascular outcomes. Besarab et al. illustrated that a higher hematocrit level (up to 42) by epoetin did not provide the benefit of lowering myocardial infarction in patients with hemodialysis [22]. The clinical trials demonstrated that the ESA increased hemoglobin, but the higher Hb target was associated with thromboembolic events and higher risks of cardiovascular outcomes, either for dialysis-dependent or non-dialysis-dependent CKD subjects [8,23,24], and the patients with ESA hyporesponsiveness were associated with higher incidences of mortality or cardiovascular events [25]. The excessive plasma erythropoietin concentration also reflected the accumulated dose of the erythropoietin [26]. The EPO hypo-responsiveness also reflected the chronic inflammation or dysregulation of iron metabolism [27]. In contrast, HIF-PHI provided a different mechanism for renal anemia. HIF-PH inhibitor alleviated the degradation of HIF-1 alpha and cooperated with the endogenous EPO production from the bone marrow cells [13]. Phase-3 clinical trials of HIF-PHI have been conducted in dialysis-dependent and independent CKD patients [28–30]. In the real-world Japanese surveillance, roxadustat demonstrated clinical efficacy in patients with NDD-CKD anemia, with over half of patients achieving hemoglobin targets within 12 weeks (54.6%). The incidence of adverse drug reactions (ADRs; 19.4%) and serious ADRs (10.1%) was lower than that observed in dialysis-dependent populations, consistent with the expectation that NDD patients have a lower comorbidity burden [31]. Similarly, interim results from the VIOLET vadadustat surveillance highlighted its favorable safety and tolerability in the NDD cohort (*n* = 1429). ADRs occurred in 14.1% and serious ADRs in 5.7%, again lower than in dialysis populations. The most frequent ADRs were gastrointestinal (nausea and diarrhea), and ADRs of special interest (malignancy 1.1%, thromboembolism 1.0%) remained within the expected range for CKD. Effectiveness analyses showed mean hemoglobin increases of +0.30 g/dL in ESA-switch patients and +1.12 g/dL in ESA-naïve patients at 12 months, with the greatest improvements seen in those with baseline Hb <10 g/dL [32]. These real-world findings are consistent with phase 3 data from PRO2TECT, where vadadustat was non-inferior to darbepoetin alfa for maintaining hemoglobin in NDD patients [30] and with earlier phase 2 trials that demonstrated robust dose-dependent Hb increases [33]. From the clinical trials of vadadustat, daprodustat, and roxadustat, the HIF-PHI provided comparable efficacy in maintaining hemoglobin levels in dialysis patients. The cardiovascular outcome, such as MACE or stroke, was similar between the HIF-PH inhibitors and the ESA among dialysis or PD patients [34–36]. Our data demonstrated that the HIF-PH inhibitor users were associated with benefits in overall mortality in CKD stage IV when compared with ESA, which was different from the outcomes from clinical trials. From the meta-analysis, the mortality and the MACE were similar based on the current clinical trials [30,37,38]. The mean GFR was approximately 20 mL/min/ m^2^ among the non-dialysis CKD trials, and the hemoglobin was approximately 9–10 g/dL. Therefore, we selected the subjects with GFR 15–30 mL/min/1.73 m^2^ for further investigation. The GFR and hemoglobin values of our current study were similar to those of other clinical trials. Therefore, the HIF-PH inhibitor might be beneficial for anemic patients with moderate chronic kidney disease. The data illustrated that the HIF-PH inhibitor was associated with a lower risk of sepsis when compared to the ESA user. Among the subgroup analyses, the subjects with a history of diabetes mellitus, using SGLT2i and ARB, had a lower risk of HIF PH inhibitors. HIF plays a key role in regulating cellular metabolism and facilitating adaptation to stress induced by oxygen deficiency. Hypoxia potentiates the antimicrobial activity by enhancing HIF-related transcription [39]. Johnson et al. demonstrated that the expression of HIF also facilitates bactericidal capacity in the phagocytic cells [40]. HIF activation in the inflamed intestinal mucosa can occur through various mechanisms, including reduced oxygen supply due to vascular damage or ischemia, as well as increased oxygen demand resulting from the heightened consumption by transmigrating neutrophils and eosinophils, both of which contribute to mucosal hypoxia [41]. Additionally, cytokine signaling can enhance HIF-1α transcription, while extracellular purinergic signaling involving neutrophils and platelet-derived ATP and Ap3A also plays a role [42,43]. Furthermore, components of the microbiome can drive HIF activation by increasing epithelial oxygen consumption or inducing iron chelation [41]. From the INNO_2_VATE trial, vadadustat in patients with peritoneal dialysis, the infection rate was lower in the HIF-PH inhibitor users (55.3% for the HIF-PH group and 69.4% for the ESA group) [44]. Since CKD patients are vulnerable to the occurrence of infection and the associated mortality, the use of HIF-PH inhibitors might provide clinical implications based on the potential for infection. To compare the HIF-PHI with long-or short-acting ESA based on the meta-analysis by Minutolo et al. we performed the comparison in October 2025 with lower case counts changed between analyses because several TriNetX HCOs that contributed data were temporarily unavailable during the current period. Consistent with the meta-analysis by Minutolo et al. which found no overall mortality difference between HIF-PHIs and ESAs, individual trials reported non-significant trends toward lower mortality (HR: 0.33 from Akizawa et al. [45] and HR: 0.49 from Chertow et al. [30]. Our real-world design distinguishes short-acting from long-acting ESA exposure and shows benefit only versus short-acting ESA, with no difference versus long-acting ESA after refined PSM. We did not analyze the combination or sequential ESA/HIF-PHI use. Longer follow-up and broader HIF-PHI utilization may provide additional clarity.

Our data demonstrated that the ferritin level did not differentiate the efficacy when setting the cutoff value at 300 ug/L. Serum ferritin levels of 100 μg/L in non-dialysis CKD patients and 200 μg/L in dialysis patients are commonly used as cutoff values [46]. Hyperferritinemia does not always accurately reflect total body iron storage or overload but may indicate an active inflammatory state. Besides, the interaction between the parameters indicating iron deposition and the epoetin dosing. VychytIil et al. illustrated that the serum ferritin level was inversely associated with the accumulated doses of epoetin. However, the database could not provide the accumulated doses of erythropoietin. Therefore, the variation between the dosing and ferritin might not be reflected. Excess iron can become trapped in macrophages, impairing phagocytic function and weakening innate immunity. Our data showed no significant difference in efficacy when setting the ferritin cutoff between 299 and 300 ng/mL. However, when comparing subjects with ferritin <100 ng/mL and those with 101–299 ng/mL, HIF-PH inhibitor users had a lower risk of infection and mortality, suggesting potential benefits of maintaining lower ferritin levels. Iron management strategies also play a critical role [47,48]. CKD significantly increases the risk of infections and sepsis due to immune dysfunction, comorbidities, and frequent invasive procedures. Infections are a major cause of mortality in these patients, particularly those on dialysis [49]. While preventive strategies such as vaccinations, improved hygiene, and catheter care have been proposed, their effectiveness remains uncertain. The complex interplay of immune impairment and inflammation in CKD necessitates further research to establish clear preventive guidelines. Despite some promising interventions, a definitive strategy to reduce infection-related complications in CKD patients is still lacking. Unlike ESA, HIF-PH inhibitors are associated with lower ferritin levels, potentially reducing persistent or episodic hyperferritinemia [50]. This raises the need to reconsider ferritin targets when using HIF-PH inhibitors. While these findings highlight a possible advantage of lower ferritin levels, larger-scale clinical trials are necessary to establish long-term effects and optimal ferritin thresholds in CKD and dialysis patients.

This study has several limitations. First, the retrospective study design lacks the rigor of randomized controlled trials, and a large RCT will ultimately be required to confirm these findings. Secondly, the majority of the subjects studied in our study originated from the APAC collaborative network. The low proportion of patients receiving HIF-PHIs in our dataset (1.3%) reflects their recent approval and restricted reimbursement in clinical practice during the study period for non-dialysis patients [51]. Based on the inconsistency of the FDA approval among different countries, the efficacy among the Caucasians or other ethnicities could not be identified [51]. Although the findings were derived from a U.S.-based healthcare setting, the study population was predominantly composed of non-White individuals. Given this demographic composition, the data may, in part, reflect populations more representative of institutions outside the United States, including those in Asia. This raises the possibility that the observed outcomes may apply to broader international contexts, particularly within Asian healthcare systems. Although propensity score matching was applied, residual confounding from unmeasured factors like socioeconomic status, nutritional condition, detailed iron treatments, or inflammation levels could still affect outcomes. At the same time, we used a hemoglobin level less than 9 g/dL as the exclusion criterion instead of iron deficiency anemia. Moreover, reliance on electronic health records may lead to incomplete data or misclassification biases. The medication adherence and exact treatment durations were not evaluable, potentially influencing comparative effectiveness. Therefore, we were unable to distinguish cases involving concurrent or sequential use of HIF-PHIs and ESAs. Our real-world dataset did not provide sufficient information on ESA dosing strategies or the selection process for dose adjustment, which limits direct comparison. Moreover, we were unable to distinguish between short- and long-acting ESA formulations, despite evidence that their efficacy in achieving Hb targets may differ. Besides, the iron deficiency anemia was not listed as an exclusion criterion. Therefore, the underlying illness worsening the RBC production might influence the outcomes. Hemoglobin response is largely determined by the administered dose rather than intrinsic drug potency. In a recent meta-analysis of randomized controlled trials [20], HIF-PHIs were associated with only a modest increase in Hb (∼0.1 g/dL) compared with ESAs at trial-specified doses. Lastly, the relatively short follow-up restricts assessment of long-term safety and effectiveness. Prospective studies with extended observation periods are required to validate these results and clarify the long-term risks associated with HIF-PH inhibitors.

## Conclusion

In this real-world propensity-matched cohort of stage IV non-dialysis chronic kidney disease patients, treatment with HIF-PHIs was associated with significantly lower risks of all-cause mortality and sepsis compared with ESAs. However, this survival benefit was confined to comparisons with short-acting ESAs; no significant difference was found between HIF-PHIs and long-acting ESAs such as darbepoetin or methoxy polyethylene glycol-epoetin beta. The advantage was most evident in patients with intermediate ferritin levels (100–299 ng/mL). These results indicate that the favorable outcomes of HIF-PHIs arise mainly from differences versus short-acting ESA formulations rather than class-wide effects. Prospective randomized trials are warranted to confirm these findings and to further define optimal iron and hemoglobin targets for both short- and long-acting erythropoiesis-stimulating regimens in non-dialysis CKD.

## Supplementary Material

Supplement_2_HIF_vs_ESA_Survival_Results_20251116.docx

Supplement_1_HIF_20251116.docx

## Data Availability

The data used in this study were obtained from the TriNetX platform, a global health research network. Due to data-use agreements and privacy restrictions, the datasets cannot be publicly shared. Researchers interested in accessing the data may contact TriNetX ([website or email]) for more information.
