# Supplementary material for: Comparative effectiveness of hypoxia-inducible factor prolyl hydroxylase inhibitors versus erythropoiesis-stimulating agents on prognosis in non-dialysis chronic kidney disease: a propensity-matched cohort study
Source: Ren Fail. 2025 Dec 4;47(1):2592442. doi: 10.1080/0886022X.2025.2592442 (PMC12679846; doi:10.1080/0886022X.2025.2592442)
Supplement: Supplement_2_HIF_vs_ESA_Survival_Results_20251116.docx [file IRNF_A_2592442_SM4942.docx]

## Supplementary Table 2. Comparative Survival Outcomes Between HIF-PHI Users and ESA Subgroups (Short-acting vs Long-acting ESA) After Propensity-Score Matching (eGFR 15–30 mL/min/1.73 m²).

| Comparison | Patients Before Matching (HIF / ESA) | Patients After Matching (HIF / ESA) | Patients with Outcome (HIF / ESA) | Survival Probability at End of Time Window | p-value (Log-Rank) | Hazard Ratio (95 % CI) |
| --- | --- | --- | --- | --- | --- | --- |
| HIF-PHI vs Overall ESA | 154 / 42,626 | 151 / 151 | 21 / 39 | 65.19 % / 57.13 % | 0.0063 | 0.533 (0.318 – 0.893) |
| HIF-PHI vs Short-acting ESA | 154 / 29,188 | 140 / 140 | 20 / 40 | 65.93 % / 57.05 % | 0.0304 | 0.554 (0.322 – 0.953) |
| HIF-PHI vs Long-acting ESA | 154 / 10,994 | 149 / 149 | 21 / 26 | 63.75 % / 65.18 % | 0.5965 | 1.172 (0.651 – 2.111) |

**Abbreviations:** HTN, hypertension; DM, diabetes mellitus; ACEi, angiotensin-converting enzyme inhibitor; ARB, angiotensin receptor blocker; CI, confidence interval; HR, hazard ratio.
