# Supplementary material for: Comparative effectiveness of hypoxia-inducible factor prolyl hydroxylase inhibitors versus erythropoiesis-stimulating agents on prognosis in non-dialysis chronic kidney disease: a propensity-matched cohort study
Source: Ren Fail. 2025 Dec 4;47(1):2592442. doi: 10.1080/0886022X.2025.2592442 (PMC12679846; doi:10.1080/0886022X.2025.2592442)
Supplement: Supplement_1_HIF_20251116.docx [file IRNF_A_2592442_SM4941.docx]

| **Items** | **Coding** |
| --- | --- |
| Death | Deceased |
| ST-segment elevation myocardial infarction | I21.3 |
| ischemic stroke | I63 |
| hemorrhagic stroke | I61 |
| Heart failure | I50 |
| sepsis | ICD-10 with A41.89, A41.9, A41.5, A41.51, A41.0, A41.01, A41.02, A41.1, A41.52, A41.81, A41.4, A41.2, T81.44XA, A40.0, A40.1, A41.53, A41.3, A41.54, R65.20, A41 or R65.2 |
| Cognitive impairment | ICD-10 with F03, F02, F22, G30, F05, G20 or F01 |
| fracture | ICD-10 with S22, S32, S42, S52, S62, S72, S82, S92 or S12 |
| MACE | ICD-10 withI63, I50, I25.2, I21.4, I22, I21.A9, I21.9 or I73.9 |
| neoplasm | ICD-10 with C15–C26, C50, C64–C68, C60–C63, C51–C58, C45–C49, C30–C39, C43–C44, C81–C96, C00–C14, C73–C75 or C76–C80 |
| Retinopathy | ICD-10 with H34, H33, H36 or H35 |
| Renal Replacement therapy | CPT with 1012740 or 90945; ICD-10 with Z99.2 or N18.6 or Z94.0 |
| HIF-PHI user | Daprodustat, vadadustat or roxadustat |
| ESA user | epoetin alfa, darbepoetin alfa, Injection, darbepoetin alfa, 1 microgram (non-esrd use), Injection, darbepoetin alfa, 1 microgram (for esrd on dialysis) or methoxy polyethylene glycol-epoetin beta |

**Supplementary Table 1. ICD-10-CM and CPT coding definitions for clinical outcomes, comorbidities, and medication exposure used in the analysis.**

| **Items** | **Coding** |
| --- | --- |
| Death | Deceased |
| STEMI | I21.3 |
| ischemic stroke | I63 |
| hemorrhagic stroke | I61 |
| CHF | I50 |
| sepsis | ICD-10 with A41.89, A41.9, A41.5, A41.51, A41.0, A41.01, A41.02, A41.1, A41.52, A41.81, A41.4, A41.2, T81.44XA, A40.0, A40.1, A41.53, A41.3, A41.54, R65.20, A41 or R65.2 |
| Cognitive impairment | ICD-10 with F03, F02, F22, G30, F05, G20 or F01 |
| fracture | ICD-10 with S22, S32, S42, S52, S62, S72, S82, S92 or S12 |
| MACE | ICD-10 withI63, I50, I25.2, I21.4, I22, I21.A9, I21.9 or I73.9 |
| neoplasm | ICD-10 with C15–C26, C50, C64–C68, C60–C63, C51–C58, C45–C49, C30–C39, C43–C44, C81–C96, C00–C14, C73–C75 or C76–C80 |
| Retinopathy | ICD-10 with H34, H33, H36 or H35 |
| HIF-PH group | Daprodustat, vadadustat or roxadustat |
| ESA group | epoetin alfa, darbepoetin alfa, Injection, darbepoetin alfa, 1 microgram (non-esrd use), Injection, darbepoetin alfa, 1 microgram (for esrd on dialysis) or methoxy polyethylene glycol-epoetin beta |
